# The prognostic value of integration of pretreatment serum amyloid A (SAA)–EBV DNA (S-D) grade in patients with nasopharyngeal carcinoma

**DOI:** 10.1186/s40169-019-0252-7

**Published:** 2020-01-06

**Authors:** Jianpei Li, Changchun Lai, Songguo Peng, Hao Chen, Lei Zhou, Yufeng Chen, Shulin Chen

**Affiliations:** 1 State Key Laboratory of Oncology in South China, Collaborative Innovation Center for Cancer Medicine, Guangdong Key Laboratory of Nasopharyngeal Carcinoma Diagnosis and Therapy, Sun Yat-sen University Cancer Center, 651 Dongfeng Road East, Guangzhou, 510060 People’s Republic of China; 2 Department Of Clinical Laboratory, Maoming People’s Hospital, Maoming, 525000 Guangdong People’s Republic of China; 3 Department Of Clinical Laboratory, The Traditional Chinese Medical Hospital of Gaozhou City, Maoming, 525000 Guangdong People’s Republic of China

**Keywords:** Nasopharyngeal carcinoma, SAA, EBV DNA, Prognostic

## Abstract

**Background:**

Serum amyloid A (SAA) has been associated with the development and prognosis of cancer. The purpose of this study was to evaluate the predictive value of integration of pretreatment SAA–EBV DNA (S-D) grade and comparison with the TNM staging system in patients with nasopharyngeal carcinoma (NPC). The S-D grade was calculated based on the cut-off values of serum SAA and EBV DNA copy numbers which were determined by receiver operating characteristic (ROC) curves. We evaluated the prognostic value of pretreatment SAA, EBV DNA and S-D grade on overall survival (OS) of NPC patients. We also evaluated the predictive power of S-D grade with TNM staging system using 4 indices: concordance statistics (C-index), time-dependent ROC (ROCt) curve, net reclassification index (NRI) and integrated discrimination improvement (IDI).

**Results:**

A total of 304 NPC patients were enrolled in this study. Multivariate analysis showed that TNM stage (P = 0.007), SAA (P = 0.013), and EBV DNA (P = 0.033) were independent prognostic factors in NPC. The S-D grade was divided into S-D grade 1, S-D grade 2, and S-D grade 3, which had more predictive accuracy for OS than TNM staging according to all 4 indices.

**Conclusions:**

We found that the S-D grade could be used as a new tool to predict the OS in NPC patients.

## Background

Nasopharyngeal carcinoma (NPC) is one of the most common malignant tumors in Southern China and Southeast Asia, with an incidence rate of 2.8 in 100,000 people per year in men and 1.9 in 100,000 people per year in women [1]. Patients diagnosed with early NPC (stages I–II) have excellent outcomes, with 5-year survival rates of up to 94%, but patients diagnosed with NPC in stages III–IV have a poorer prognosis, whith 5-year survival rates lower than 80% [2]. Currently, the general standard for predicting prognosis and facilitating treatment stratification of NPC patients is the Union Internationale Contre le Cancer/American Joint Cancer Committee (UICC/AJCC) TNM staging system [3, 4]. This staging system only focuses on the tumor size, extension and node involvement, and does not consider other prognostic factors (clinicopathologic features, treatment- related factors, inflammatory state). As a result, the NPC patients with similar histologic classifications and stages often have very different survival outcomes, identifying need for a more precise method to improve the prediction of NPC patient outcomes.

Chronic inflammation is a key contributor to cancer initiation, promotion, progression, and metastasis [5]. Serum amyloid A (SAA) is a nonspecific, acute-phase, hepatic protein secreted in response to cytokines [6]. It is also an HDL-associated lipoprotein known to play a major role as a modulator of inflammation and in the metabolism and transport of cholesterol [7]. Of interest, SAA has been reported as a potentially prognostic biomarker in many cancers including renal cancer, lung cancer, melanoma, esophageal squamous cell carcinoma, breast cancer, and hepatocellular carcinoma [8–13]. Epstein-Barr virus (EBV) infection plays an important role in NPC pathogenesis [14]. The plasma EBV DNA assay is widely used for screening, prognostic prediction, and post-treatment surveillance of patients with NPC [15].

At present, no published studies have combined SAA and EBV DNA as predictive markers for overall survival in NPC patients. The aim of this study was to investigate the prognostic value of integration of pretreatment SAA–EBV DNA (S-D) grade in NPC patients. These results will provide a simple and precise prognostic tool to predicting survival in NPC patients.

## Methods

### Patients and study design

We performed a retrospective analysis of NPC patients were treated at Sun Yat-sen University Cancer Center (Guangzhou, China) between December 2008 and December 2011. Patients were enrolled in this study if they met the following inclusion criteria: (1) NPC diagnosis confirmed by histopathology, no other malignancies; (2) data were obtained prior to anti-tumor treatment; (3) complete baseline clinical information and laboratory data; (4) complete follow-up data; (5) death resulting from cancer-specific death.

Clinical data were collected from medical records prior to anti-tumor treatment, including age, gender, TNM stage, therapeutic data, C-reactive protein (CRP), Serum amyloid A (SAA) level, platelet-to-lymphocyte ratio (PLR), neutrophil-to-lymphocyte ratio (NLR), lymphocyte-to-monocyte ratio (LMR), and EBV DNA copy number. Clinical stage was classified according to the 7th TNM classification of American Joint Committee on Cancer (AJCC) staging manual [16].

All patients provided written informed consent for enrollment in this research study. This study was approved by the Hospital Ethics Committee in Sun Yat-sen University Cancer Center in China. The authenticity of this article has been validated by uploading the key raw data onto the Research Data Deposit public platform (http://www.researchdata.org.cn), with the approval RDD number as RDDA2019001145.

### Clinical outcome assessment and patient follow-up

The patients were followed-up by telephone, letter, or at an outpatient interview. Our endpoint was cancer-specific overall survival (OS), which was defined as the interval between the date of NPC diagnosis to the date of death due to malignancy, or by patient censoring on the date of last follow-up. All patients were followed until death or until August 2015 (end of study).

### Construction of SAA–EBV DNA (S-D) grade

According to the Youden index by receiver operating characteristic (ROC) curve, the cut-point for SAA was 4.46 mg/L, and for EBV DNA was 2340 copies/mL. Based on these cut-off values, the prognostic value of S-D grade was defined as follows: S-D grade 1: patients with both a SAA level ≤ 4.46 mg/L and EBV DNA ≤ 2340 copies/mL; S-D grade 2: if either of the two markers were elevated; S-D grade 3: patients with both markers elevated.

### Statistical analysis

Statistical analysis was performed using SPSS ver. 19.0 (SPSS Inc., Chicago, IL, USA) and R version 3.6.0. Categorical variables were stratified by clinical application, and the optimum cut-off points of continuous variables for predicting the overall survival (OS) were determined by receiver operating characteristic (ROC) analyses. Survival curves were plotted using the Kaplan–Meier (KM) method and compared using log-rank test. Variables with P < 0.05 in the univariate analysis were entered into Cox proportional hazards analyses. Independent prognostic factors were determined with multivariate Cox analyses (P < 0.05). The discrimination ability of SAA, EBV DNA, S-D grade and TNM staging system to predict survival were measured by Harrell’s [17] concordance index (C-index), time-dependent receiver operative characteristics (ROCt) [18], net reclassification index (NRI) [19] and integrated discrimination improvement (IDI) [19]. The C-index was defined as the proportion of patient pairs in which the predicted and observed survival outcomes were concordant. Time-dependent ROC (ROCt) curves by plotting sensitivity versus specificity, and areas under the curve (AUC) were calculated to estimate the predictive accuracy. The NRI was a new statistical method to measure the improvement in predictive performance of a new model to re-classify subjects compared to an old model into binary event or no-event categories [20]. The NRI assigned a numerical score of + 1 for upward reclassification, − 1 for downward and 0 for subjects who were not reclassified. Individual scores were summed separately for the event and non-event groups, and divided by numbers of subjects in each group. The IDI was computed based on integrated sensitivity and specificity which can be defined as a difference in discrimination slope between an existing model and a new model [21]. If the IDI > 0, this indicated that the new model had better prediction ability than the old model. If the IDI < 0, it was negative improvement, and the new model’s prediction ability was less than the old model. If IDI = 0, both models were able to predict survival with equal strength. Overall, larger values for the C-index, AUC, NRI and IDI indicated more accurate prognostic stratification. A two-sided P value of < 0.05 was considered statistically significant for all analyses performed.

## Results

### Patient characteristics and cutoff Values of continuous variables

The clinicopathologic characteristics of patients were shown in Table 1. A total of 304 patients with a pathological diagnosis of NPC were enrolled in this study, with 232 (76.3%) male and 72 (23.7%) female patients. The median age was 46 years (range, 17–78 years), with a median follow-up time of 44.4 months (range 0.7–76).

**Table 1:** Clinical and laboratory characteristics of 304 patients associated with overall survival (OS)

Patient characteristics	No. of patients (%)	Median OS (IQR)	P-value^a^
Gender			
Male	232 (76.3%)	44.2 (38.5–47.4)	0.967
Female	72 (23.7%)	44.8 (40.7–46.8)	
Age (years)			
≤46	157 (51.6%)	45.0 (40.6–47.8)	0.202
> 46	147 (48.4%)	43.4 (37.2–46.8)	
Tumor stage			
T1–2	52 (17.1%)	46.2 (41.9–49.8)	0.043
T3–4	252 (82.9%)	44.0 (38.3–46.9)	
Node stage			
N0–1	168 (55.3%)	44.5 (40.8–47.0)	0.007
N2–3	136 (44.7%)	43.9 (36.0–47.8)	
TNM stage^b^			
I–II	33 (10.9%)	45.8 (41.3–48.0)	0.048
III–IV	271 (89.1%)	44.2 (39.2–47.1)	
Treatment			
Radiotherapy	43 (14.1%)	44.0 (40.7–48.1)	0.047
Chemoradiotherapy	261 (85.9%)	44.4 (38.7–47.0)	
CRP (mg/L)			
≤ 2.03	131 (43.1%)	44.6 (41.9–47.4)	0.001
> 2.03	173 (56.9%)	44.1 (36.0–47.0)	
SAA (mg/L)			
≤ 4.46	154 (50.7%)	44.7 (41.7–47.4)	0.001
> 4.46	150 (49.3%)	43.3 (35.2–47.1)	
PLR			
≤ 141.52	167 (54.9%)	44.0 (40.1–47.0)	0.287
> 141.52	137 (45.1%)	45.1 (37.2–47.5)	
NLR			
≤ 2.62	149 (49.0%)	44.2 (40.6–47.5)	0.006
> 2.62	155 (51.0%)	44.5 (36.1–47.0)	
LMR			
≤ 1.87	29 (9.5%)	45.3 (42.1–50.3)	0.415
> 1.87	275 (90.5%)	44.2 (39.2–47.0)	
EBV DNA (copies/mL)			
≤ 2340	161 (53.0%)	45.0 (40.9–47.6)	<0.001
> 2340	143 (47.0%)	43.4 (36.0–46.4)	

^a^Survival was analyzed using Kaplan–Meier method and compared with Log-Rank test;

^b^TNM stage was classified according to the AJCC 7th TNM staging system;

*IQR* interquartile range, *TNM* tumor node metastasis stage, *CRP* C-reactive protein, *SAA* serum amyloid A, *PLR* platelet/lymphocyte ratio, *NLR* neutrophil/lymphocyte ratio, *LMR* ymphocyte/monocyte ratio, *EBV* Epstein-Barr virus

According to the ROC curves, the optimal cut-off points for age, CRP, SAA, PLR, NLR, LMR, and EBV DNA were 46, 2.03, 4.46, 141.52, 2.62, 1.87, and 2340, respectively.

### Univariate and multivariate survival analysis

The results of the univariate and multivariate analysis for OS were shown in Table 2. Univariate analysis demonstrated that node stage (P = 0.008), TNM stage (P < 0.001), CRP (P = 0.002), SAA (P = 0.001), and EBV DNA (P = 0.01) were prognostic factors in NPC patients. Multivariate Cox-analysis showed that TNM stage [hazard ratio (HR): 2.190, 95% confidence interval (CI) 1.234–3.866, P = 0.007), SAA (HR: 2.276, 95% CI 1.186–4.368, P = 0.013), and EBV DNA copy number (HR: 2.075, 95% CI 1.061–4.060, P = 0.033) were independent prognostic factors in these patients.

**Table 2:** Univariate and multivariate analysis for overall survival (OS) for the 304 patients with NPC

Variables	Univariate analysis			Multivariate analysis^a^		
	HR	95.0% CI for HR	*P* value	HR	95.0% CI for HR	P-value
Gender						
Male vs female	1.014	0.515–1.997	0.967	–	–	–
Age						
≤46 years vs > 46 years	1.458	0.814–2.612	0.205	–	–	–
Tumor stage						
T1–2 vs T3–4	3.17	0.976–10.144	0.055	–	–	–
Node stage						
N0–1 vs N2–3	1.497	1.110–2.020	0.008	–	–	–
TNM stage						
I vs II vs III vs IV	2.971	1.709–5.165	<0.001	2.190	1.234–3.886	0.007
Treatment						
Radiotherapy vs chemoradiotherapy	3.800	0.921–15.674	0.065	–	–	–
CRP (mg/L)						
≤ 2.03 vs > 2.03	3.014	1.496–6.074	0.002	–	–	–
SAA (mg/L)						
≤ 4.46 vs > 4.46	2.956	1.555–5.617	0.001	2.276	1.186–4.368	0.013
PLR						
≤ 141.52 vs > 141.52	1.368	0.767–2.440	0.289	–	–	–
NLR						
≤ 2.62 vs > 2.62	2.361	1.260–4.426	0.007	–	–	–
LMR						
≤ 1.87 vs > 1.87	1.619	0.502–5.219	0.420	–	–	–
EBV DNA (copies/mL)						
≤ 2340 vs > 2340	3.079	1.620–5.851	0.001	2.075	1.061–4.060	0.033

^a^Variables with P < 0.05 in the univariate analysis (Node stage, TNM stage, CRP, SAA, NLR, and EBV DNA) were entered into multivariate analysis

### Survival Analysis of SAA, EBV DNA, and S-D grade

**Fig. 1:**
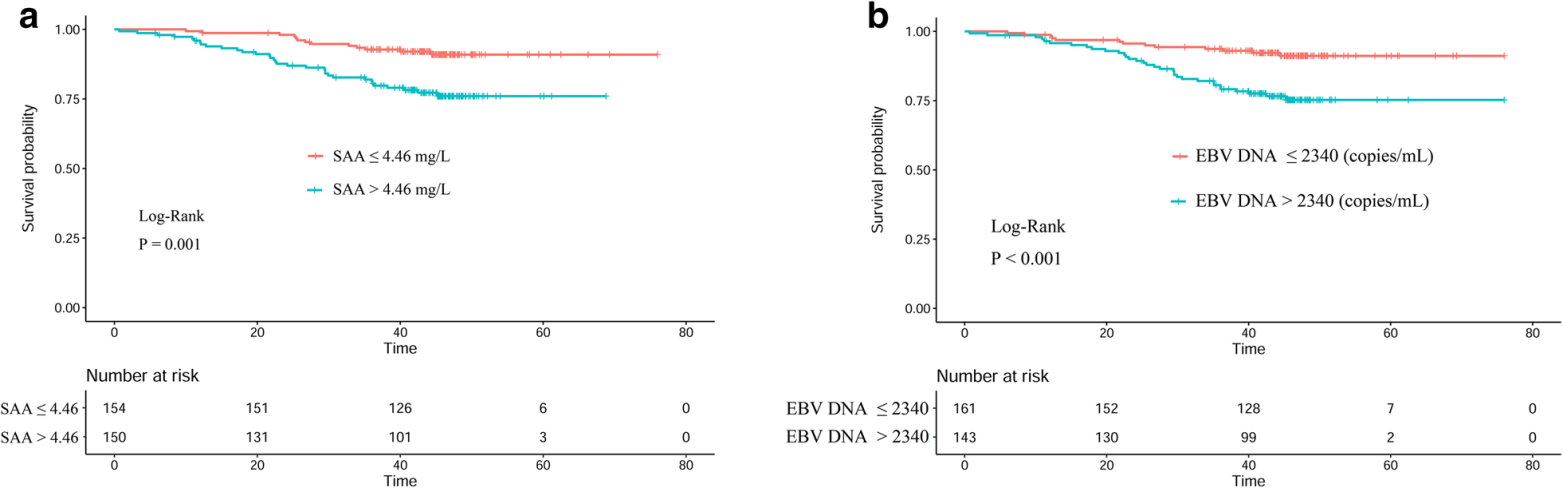
Kaplan–Meier curves of SAA and EBV DNA for OS in NPC patients

**Fig. 2:**
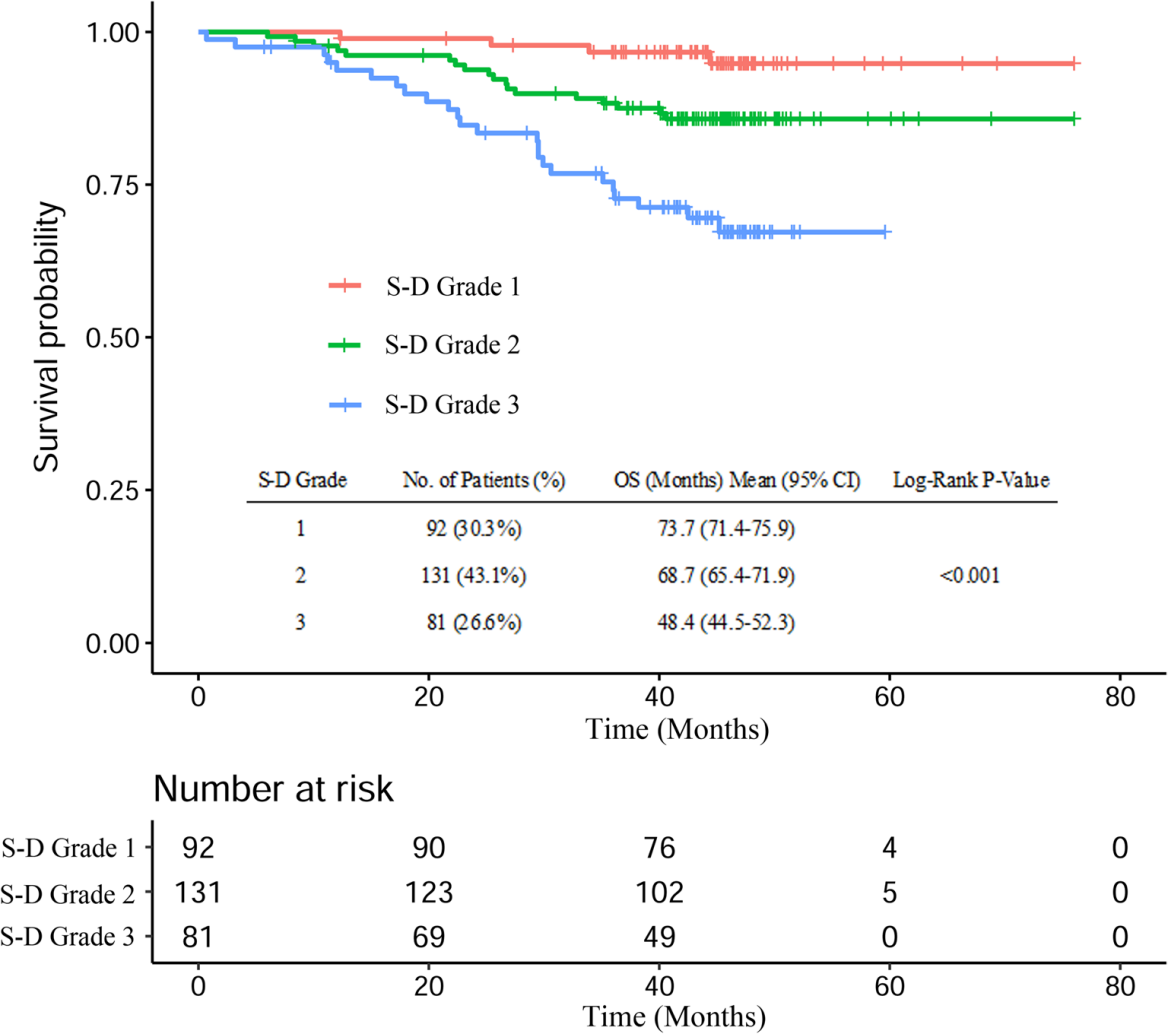
Kaplan–Meier curves of S-D grade for OS in NPC patients

Kaplan–Meier analysis demonstrated that the survival of patients was significantly different when SAA (P = 0.001, Fig. 1a) or EBV DNA (P < 0.001, Fig. 1b) was used. For the patients with S-D grade 1, the median survival time was 45.2 (IQR: 41.7–47.6) months, which was higher than the S-D grade 2 (44.6 months, IQR: 40.3–47.3) and S-D grade 3 (48.4 months, IQR: 29.5–46.9). The OS of patients with S-D grade 1 was also higher than those with S-D grade 2 or S-D grade 3 (P < 0.001, Fig. 2).

### Comparison of prognostic accuracy of SAA, EBV DNA, S-D grade and TNM stage using c-index and time-dependent ROC analysis

**Fig. 3:**
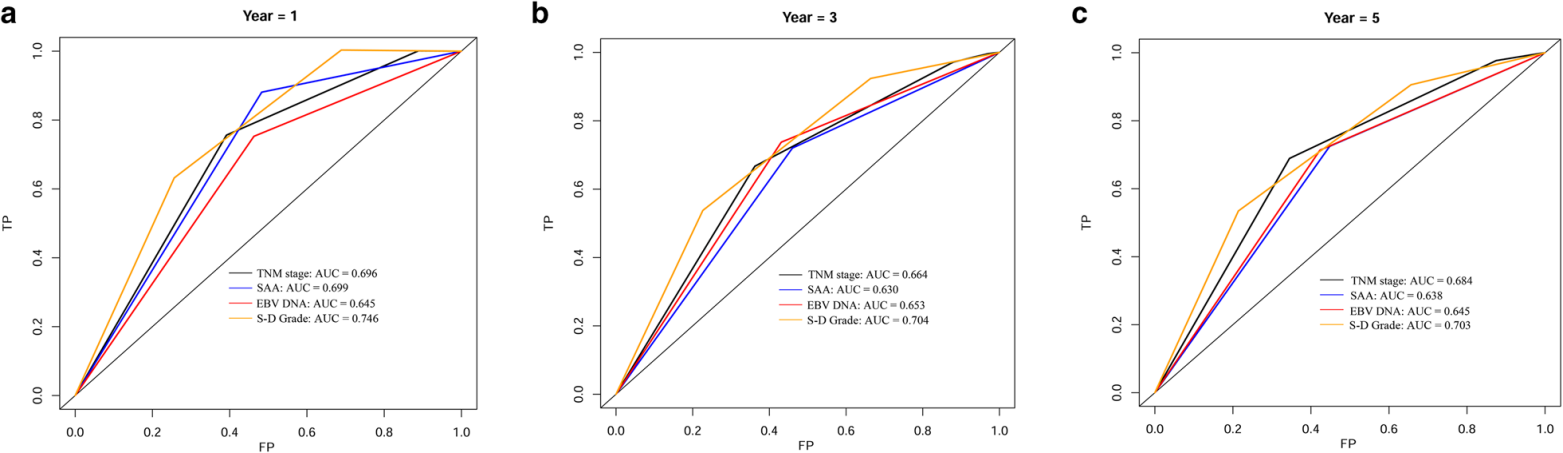
Time-dependent ROC analysis of TNM stage, SAA, EBV DNA, and S-D grade for 1-, 3-, and 5-year OS in NPC patients

The C-index for SAA, EBV DNA, S-D grade and TNM stage was 0.631, 0.633, 0.689 and 0.656, respectively (Table 3). The S-D grade showed increasing prognostic accuracy compared with TNM stage, but there was no statistically significant differences between these two classifiers. Results for time-dependent ROC analysis showed similar findings for 1-, 3-, and 5-year survival, with the AUC of S-D grade has the highest among the four assessment methods (Fig. 3). These results indicated that the S-D grade can better predict outcomes than TNM stage.

**Table 3:** The C-index of SAA, EBV DNA, S-D grade and TNM stage for prediction of OS

Factors	C-index (95% CI)	△C-index^a^	*P*
SAA	0.631 (0.565–0.696)		
EBV DNA	0.633 (0.566–0.700)		
S-D grade	0.689 (0.623–0.754)		
TNM stage	0.656 (0.590–0.723)		
S-D grade vs SAA		0.058	0.062
S-D grade vs EBV DNA		0.056	0.030
SAA vs TNM stage		− 0.025	0.568
EBV DNA vs TNM stage		− 0.023	0.530
S-D grade vs TNM stage		0.032	0.441

Statistic indicate the changes in C statistic

*C-index* concordance index, *CI* confidence interval, *S-D grade* SAA-EBV DNA grade

### Comparison of discriminatory ability of SAA, EBV DNA, S-D grade and TNM stage using NRI and IDI

Results for NRI and IDI analyses at 1-, 3- and 5-year using the SAA, EBV DNA, S-D grade and TNM staging system were shown in Table 4. For NRI at 1-, 3- and 5-year survival, the discriminatory ability of S-D grade was increased 12.4%, 9.6% and 11.6%, respectively (P > 0.05), compared with TNM stage. For 1-, 3- and 5-year survival, the discriminatory ability of S-D grade was increased 0.4%, 1.8% and 3.8%, respectively (P > 0.05) compared to IDI. These results indicated that the S-D grade had superior discriminatory ability to predict survival over the TNM stage.

**Table 4:** A comparison of discriminatory ability of SAA, EBV DNA and SAA + EBV DNA with TNM stage using NRI and IDI^a^

	1-Year				3-Year				5-Year			
	NRI	P	IDI	P	NRI	P	IDI	P	NRI	P	IDI	P
SAA vs TNM stage	− 4.8%	0.641	− 0.1%	0.827	− 3.3%	0.689	− 1.4%	0.635	− 17.2%	0.515	− 3.5%	0.549
EBV DNA vs TNM stage	− 15.6%	0.482	− 0.4%	0.541	− 5.3%	0.549	− 0.8%	0.723	− 12.5%	0.615	− 1.4%	0.771
S-D grade vs TNM stage	12.4%	0.472	0.4%	0.589	9.6%	0.348	1.8%	0.571	11.6%	0.537	3.8%	0.470

NRI or IDI > 0, it was positive improvement, this indicated that the new model had better prediction ability than the old model. If NRI or IDI < 0, it was negative improvement, and the new model’s prediction ability was less than the old model. If NRI or IDI = 0, it was considered the new model had not changed

### Relationship between SAA, EBV DNA, S-D grade and clinicopathological features

The association between the SAA, EBV DNA, S-D grade and clinicopathologic characteristics were shown in Table 5. Our results demonstrated that each of the SAA, EBV DNA and S-D grade was positively correlated with the node stage (P < 0.05), TNM stage (P < 0.05) and treatment (P < 0.05).

**Table 5:** Relationship between the SAA and EBV DNA pretreatment and the clinical characteristics in 304 patients with NPC

Variables	SAA (mg/L)			EBV DNA (copies/mL)			S-D Grade			
	≤ 4.46 (n = 154)	> 4.46 (n = 150)	P	≤ 2340 (n = 161)	> 2340 (n = 143)	P	1 (n = 92)	2 (n = 131)	3 (n = 81)	P
Gender										
Male	114	118	0.349	123	109	1.000	70	97	65	0.586
Female	40	32		38	34		22	34	16	
Age (years)										
≤ 46	76	81	0.424	80	77	0.492	46	64	47	0.401
> 46	78	69		81	66		46	67	34	
Tumor stage										
T1–2	32	20	0.095	34	18	0.066	22	22	8	0.050
T3–4	122	130		127	125		70	109	73	
Node stage										
N0–1	95	73	0.028	118	50	<0.001	66	81	21	<0.001
N2–3	59	77		43	93		26	50	60	
TNM stage										
I	5	3	0.016	7	1	<0.001	5	2	1	<0.001
II	18	7		18	7		13	10	2	
III	81	68		94	55		52	71	26	
IV	50	72		42	80		22	48	52	
Treatment										
Radiotherapy	28	15	0.048	32	11	0.003	21	18	4	0.003
Chemoradiotherapy	126	135		129	132		71	113	77	

## Discussion

In this study, we found that SAA level and EBV DNA copy number were independent predictors of OS in a cohort of NPC patients. Here, we combined SAA and EBV DNA, a parameter we called S-D grade, to assess its relationship with overall survival in NPC patients, and compared the predictive power of S-D grade with traditional TNM staging systems using C-index, ROCt curve, NRI, and IDI. Our results showed that the S-D grade was better able to predict overall survival with more accuracy than the TNM staging system, which might facilitate individualized prediction for future consultation.

SAA was synthesized mainly in the liver, and its expression regulated by cytokines including interleukins 1 (IL-1) and 6 (IL-6) released from activated macrophages [22]. Over-expression of SAA has previously been reported in NPC, renal cancer, gastric cancer, hepatocellular cancer, melanoma, breast cancer, and endometrial cancers [10, 23–29]. SAA can stimulate the expression of MMP-9 by macrophages, which in turn facilitates cancer cell metastasis [30]. Potentially, SAA could promote cancer progression by inhibiting platelet adhesion and enhancing plasminogen activation, both of which were involved in extracellular matrix (ECM) degradation and tissue remodeling [31, 32]. Conversely, SAA may have a role in combating malignancies via the inhibition of cell adhesion to ECM glycoproteins [33]. SAA had also been reported to have a role in cancer prognosis in renal cell carcinoma [34, 35], lung [9, 36], breast cancer [12], esophageal squamous cell carcinoma [11] and hepatocellular carcinoma [13]. SAA level was tremendous increased at time of relapse in NPC patients, and showed potential as a useful biomarker to monitor relapse of NPC [23]. Previously, Chen et al. [37] reported that pretreatment SAA had a certain relationship with the prognosis of NPC, and patients with high levels of SAA had poor outcomes. In this study, they reported that SAA wasn’t an independent prognostic factors and they did not assess the use of integrated SAA and EBV DNA (S-D grade) in the prognosis of NPC.

A strength of this study was the combination of SAA level and EBV DNA copy number (S-D grade) in predicting the prognosis of patients with NPC, and the use of both common approaches (C-index and ROCt curve) and novel metrics (NRI and IDI) to assess discrimination ability. Each approach all showed the S-D grade had better predictive accuracy than TNM staging systems.

Of course, our study had some limitations. First, we can’t avoid potential selection biases due to the retrospective nature. Second, our data was obtained from one centre and and the lack of an independent validation cohort to assess the predictive power of S-D grade. Therefore, our results should be validated in other data sets in future study. Third, the C-index value for S-D grade of 0.689, which was not very high. We believed that the S-D grade could combine with other prognostic factors to improve the prediction of outcomes in NPC patients. In addition, more detailed study regarding the relationship between the S-D grade and the prognosis of NPC patients should be performed throughout the whole treatment period, including prior to treatment, during treatment, and following treatment.

## Conclusion

In conclusion, we reported a novel S-D grade system that can be used to predict prognosis in NPC patients. The S-D grade provided a more predictive accuracy and discriminative ability for the OS compared with the TNM staging system. In the future, it could be used to help clinicians with decision-making and guiding treatment of patients with NPC.

## Data Availability

The data of this study are available from professor Shulin Chen, the State Key Laboratory of Oncology in South China with the reasonable request.

## Abbreviations

AUC: the areas under ROC curve
CRP: C-reactive protein
C-index: concordance statistics
EBV: Epstein-Barr virus
IDI: integrated discrimination improvement
IQR: interquartile range
LMR: lymphocyte/monocyte ratio
NRI: net reclassification index
NPC: nasopharyngeal carcinoma
NLR: neutrophil/lymphocyte ratio
OS: overall survival
PLR: platelet/lymphocyte ratio
ROC: receiver operating characteristic
ROCt: time-dependent ROC curve
SAA: serum amyloid A
S-D grade: integrated SAA and EBV DNA
TNM: tumor Node Metastasis stage
